# Snowflake: visualizing microbiome abundance tables as multivariate bipartite graphs

**DOI:** 10.3389/fbinf.2024.1331043

**Published:** 2024-02-05

**Authors:** Jannes Peeters, Daniël M. Bot, Gustavo Rovelo Ruiz, Jan Aerts

**Affiliations:** ^1^ Data Science Institute, Hasselt University, Diepenbeek, Belgium; ^2^ Expertise Center for Digital Media, Hasselt University—Flanders Make, Diepenbeek, Belgium; ^3^ Visual Data Analysis Lab, Department of Biosystems, KU Leuven, Leuven, Belgium

**Keywords:** microbiome composition, taxonomy, metagenomics, visualization method, visualization application

## Abstract

Current visualizations in microbiome research rely on aggregations in taxonomic classifications or do not show less abundant taxa. We introduce Snowflake: a new visualization method that creates a clear overview of the microbiome composition in collected samples without losing any information due to classification or neglecting less abundant reads. Snowflake displays every observed OTU/ASV in the microbiome abundance table and provides a solution to include the data’s hierarchical structure and additional information obtained from downstream analysis (e.g., alpha- and beta-diversity) and metadata. Based on the value-driven ICE-T evaluation methodology, Snowflake was positively received. Experts in microbiome research found the visualizations to be user-friendly and detailed and liked the possibility of including and relating additional information to the microbiome’s composition. Exploring the topological structure of the microbiome abundance table allows them to quickly identify which taxa are unique to specific samples and which are shared among multiple samples (i.e., separating sample-specific taxa from the core microbiome), and see the compositional differences between samples. An R package for constructing and visualizing Snowflake microbiome composition graphs is available at https://gitlab.com/vda-lab/snowflake.

## 1 Introduction

Data visualization is essential for exploring the microbiome, as it has become the focus of multiple microbiome analysis tools (McMurdie and Holmes, 2013; Wang et al., 2016; McNally et al., 2018; Buza et al., 2019; Reeder et al., 2021). The microbiome is typically formatted in an abundance table that contains the absolute or relative counts of the microorganisms observed in the collected samples. These microorganisms are the result of a classification of the sequence reads into operational taxonomic units (OTU) or amplicon sequence variants (ASV) (Kuczynski et al., 2012). This classification is done up to a certain taxonomic level (domain, kingdom, phylum, class, order, family, genus, species), depending on the quality and accuracy of the sequencer and the reference database, hence introducing a hierarchical structure in the data. Based upon our previous research, we found that preliminary analyses typically explore baseline characteristics, such as the composition of microbiomes in collected samples, the (relative) abundance and variability (distribution) of observed operational taxonomic units (OTUs) or amplicon sequence variants (ASVs), and the data’s phylogenetic structure (Peeters et al., 2021).

Visualizations frequently used to display microbiome composition are (stacked) bar charts, heat maps, Venn diagrams, and tree structures (including radial trees and cladograms) (Shade and Handelsman, 2012; Sohn et al., 2014; Lupatini et al., 2017; Hallmaier-Wacker et al., 2019; Zhang et al., 2019; Chao et al., 2021; Hamad et al., 2022). Both (stacked) bar charts and heat maps provide information about the (relative) abundance of taxa within samples or cohorts. However, because these methods use a one-dimensional space to encode the enormous number of reads observed in 16s rRNA sequences, they rarely represent all reads directly. Instead, they often use aggregations in (higher level) taxonomic classifications or neglect less abundant taxa by combining them into an “others” category (Chao et al., 2021; Hamad et al., 2022). Even in terms of perception, they might not be best suited for comparing relative abundances. Comparing the length of stacked bars amongst each other can be challenging (Saket et al., 2018), and color saturation as a channel in heat maps has well-known issues for comparing non-consecutive cells (Mackinlay, 1986; Munzner, 2014). Venn diagrams provide an overview of where taxa occur in the data, distinguishing the core microbiome from the sample or cohort-specific taxa. The main disadvantage of this visualization is that it becomes ineffective when more than four categorical groups need to be displayed (Shade and Handelsman, 2012).

In the last decade, more emphasis has been put on improving the visual encoding of the microbiome, resulting in custom visualizations that can take multiple of these baseline characteristics into account. Krona (Ondov et al., 2011) uses sunburst charts in which a combination of depth and area denotes the sample’s phylogeny, and color represents relative abundance. Metacoder (Foster et al., 2017) introduces the “heat tree” to display quantitative values (e.g., abundance or pairwise differences) in the nodes and edges of the taxonomic radial tree by means of color. GraPhlAn (Asnicar et al., 2015) lets the user annotate a radial tree representing phylogenies with metadata such as community abundances and host and environmental phenotypes. Nevertheless, they all rely on taxonomic classification aggregations rather than displaying the individual OTUs or ASVs.

In this article, our objective is to contribute to the visual exploration of the microbiome by providing a visualization method called Snowflake that focuses on in-depth data exploration and can display every individual OTU or ASV captured in the samples. A visual overview of all sampled microbes enables the straightforward identification of microorganisms unique to specific samples and those shared among multiple samples. This approach provides valuable insights into the core microbiome composition. It aids in identifying candidate microorganisms common to specific sample cohorts in clinical studies (e.g., disease vs. healthy), thus enhancing our understanding of microbiome dynamics and their relevance in various research domains.

With Snowflake, we aim to provide a clear overview of the microbiome composition in collected samples without compromising on the level of detail. The strength of our design is that it displays every observed OTU/ASV in the microbiome abundance table without losing any information due to classification or neglecting less abundant reads. Snowflake supports displaying the phylogenetic structure while keeping an overview of every distinct OTU/ASV and their presence in the samples. Moreover, additional information obtained from further downstream analysis (e.g., alpha- or beta-diversity) or metadata (e.g., disease status) can be visually encoded in the visualization. Our proposed method is based on translating the tabular microbiome abundance table into a multivariate bipartite graph structure—hereafter referred to as *microbiome composition graph*—by adding relations between samples (rows) and OTUs/ASVs (columns). By showing the microbiome composition graph as a node-link diagram, differences between samples in terms of composition, richness, and diversity can conveniently be found by looking for clusters and connectivity in the topological structure. Our visualization method underwent evaluation with the ICE-T methodology (Stasko, 2014) by a group of domain experts, yielding positive feedback. Additionally, Snowflake has been implemented in an R package for broader accessibility and implementation.

## 2 Materials and methods

In this section, we will elucidate the data transformation procedure, introduce our suggested visual encoding, and present the evaluation process through which we tested our methods.

### 2.1 Data and transformation

The data used to generate the visualizations in this paper originate from a study by Vandeputte et al. (2017), in which the gut microbiome of 40 healthy adults was profiled via 16S sequencing. A subset of 10 samples was taken for illustrative purposes to create the visuals in this paper. The 16S sequencing files of these 10 samples were reprocessed into microbiome abundance tables (storing ASVs) using DADA2 (Callahan et al., 2016), without filtering on read abundance. The code for reproduction is provided in the GitLab repository (https://gitlab.com/vda-lab/snowflake).

**FIGURE 1:**
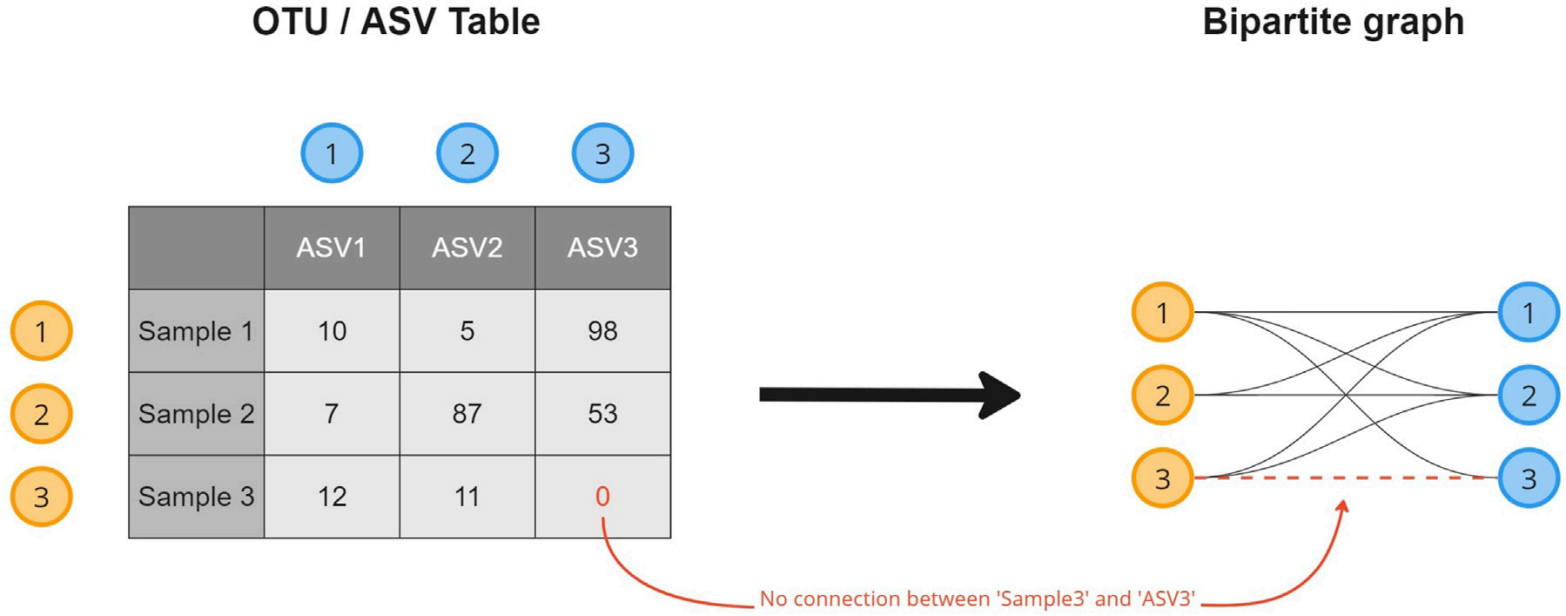
A visual overview of how a microbiome composition graph is constructed based on the tabular data format. ASV 1 is observed 10 times in sample 1, as such a relation between both is established. ASV 3 is not observed in sample 3 for which no relation will be created.

A microbiome abundance table contains the absolute or relative counts of the microorganisms (columns) observed in the collected samples (rows). To transform the data into a microbiome composition graph and create a topological overview of the presence of an OTU/ASV in the collected samples, a relational structure is introduced to the data (Figure 1). This transformation results in a bipartite graph, a common format in biological studies (Li et al., 2020; Chi et al., 2021; Calvet, 2022), where every row (sample) and column (OTU/ASV) in the table becomes an object (node) stored in a node list. Objects for which a non-zero cell value exists (abundance > 0), are linked, storing its absolute and relative abundances in a weight property. Formatting requires an edge to have a “*source*” and “*target*” property, denoting the direction of the link. The source property is reserved for the samples, and the target property is for the OTUs/ASVs. The node list contains samples and microorganisms, accompanied by all relevant information added as node attributes (Liu et al., 2011), resulting in an *attributed relational graph* structure (Weaver, 2010). Hence, baseline characteristics, as well as additional information obtained from further downstream analysis (e.g., alpha- or beta-diversity) and metadata (e.g., grouping variables), can be stored in the node attributes.

In the R package, a set of node attributes is automatically generated for both the source (samples) and target nodes (OTUs/ASVs). In the node attributes of the ASVs, we include the hierarchical structure of the taxonomic classifications, the abundance of the ASVs (col sums) in relation to the total table abundance, and the number of samples an ASV is observed in. For the samples, alpha richness- and diversity metrics (Chao1, Shannon, and Inverse-Simpson) and beta diversity metrics (Bray-Curtis, Jaccard, and Jensen-Shannon divergence) are provided as node attributes. The microbiome abundance table is therefore transformed into a directed graph without loss of information. In fact, we allow researchers to include additional information on both distinct nodes and their relationships.

### 2.2 Visual encoding

Pavlopoulos et al. (2018) surveyed the use of bipartite graphs in system biology and medicine. They discussed several visual representations for bipartite graphs: the (vertical) bipartite layout, biadjacency matrix, projected unipartite networks, and chord diagram. Misue (2006) makes use of anchored maps to draw bipartite graphs, anchoring some nodes (a certain type) to certain positions, leaving the others to move freely in the node-link diagram. In 2019, Nobre et al. (2019) conducted a survey on state-of-the-art multivariate network visualization and proposed guidance for choosing between different visual encodings. They distinguish two types of tasks—i.e., *analyzing topology for given attributes* and *analyzing attributes for a given topological structure*—and three types of encodings—i.e., node-link layouts, tabular layouts, and implicit tree layouts. Tree layouts will not be considered in this work as our primary interest is revealing the microbial composition through the topological structure of the data.

The remaining two layouts—i.e., node-link layouts and tabular layouts—have been compared in numerous studies (Ghoniem et al., 2004, 2005; Keller et al., 2006). A recent study by Okoe et al. (2019) concluded that node-link diagrams are better suited for displaying sparse networks, as they fully leverage the two-dimensional area. In addition, they found that adjacency matrices are more time-consuming and less precise for finding connections and edges between nodes. On the other hand, adjacency matrices perform better in avoiding ambiguity problems by eliminating occlusion problems in edge crossings, and it is easier to find nodes in adjacency matrices.

A special type of node-link layout designed for the visualization of bipartite graphs is the bipartite layout. Instead of the positioning of the nodes being driven by forces, source and target nodes are positioned separately on vertical axes, and lines denote their links. Hence, the link direction is always left to right (source—target). Abdelaal et al. (2022) compared this layout against the node-link diagram and adjacency matrix in terms of five tasks conducted on networks of different sizes, densities, and classes. In terms of cluster detection—the task of interest for this paper—node-link diagrams perform best in terms of accuracy and users’ assessment of task difficulty, especially in comparison with bipartite layouts. No significant differences, however, were found compared to the results of the adjacency matrix. Moreover, restricting the drawing space to only the area between the two axes makes the bipartite the least scalable among the three representations discussed by Abdelaal et al. (2022) with respect to network density.

Since our interests are both in the data’s topological structure (identifying neighbors and clusters) and in exploring the node attributes on the given topological structure, Nobre et al. (2019) favor the use of an adjacency matrix over node-link diagrams when no interactions are used. However, considering the network is sparse, has two distinct node types, and has a considerable number of nodes, topology-driven node-link layouts better suit the structure of the data (Nobre et al., 2019; Okoe et al., 2019). Therefore, we choose to work out a force-driven node-link layout but provide a possible alternative representation using an adjacency matrix.

#### 2.2.1 Node-link diagram

Our microbiome composition graph is visually encoded as a force-directed node-link diagram, following the approach used by Sedlar et al. (2016). These authors previously used bipartite graphs to visualize microbiome data. However, their focus was on the method rather than the visual representation, and they relied on aggregations of the OTUs/ASVs in their taxonomic classifications. In their proposed method, they construct six “biadjacency matrices” (one for each taxonomic level), in which links are created based on the presence of taxa in a sample at a particular taxonomic level, storing their relative abundance as weights. These are visualized using node-link diagrams in Gephi (Bastian et al., 2009) based on the ForceAtlas2 (Jacomy et al., 2014) layout algorithm. Snowflake only builds one bipartite graph storing the OTUs/ASVs taxonomy as node attributes such that every read is given equal importance and no information is lost. All nodes are represented as circles and are connected by lines if a link exists between them. Furthermore, in comparison to Sedlar et al. (2016) Snowflake includes additional information from downstream analysis in the visualization. To improve the readability of the node-link diagram, the authors advocate for the aggregation of samples into communities based on their environmental properties and to perform visual analysis at their higher taxonomic levels. In our contribution, we address this issue with a custom spatialization of the nodes in the node-link diagram.

**FIGURE 2:**
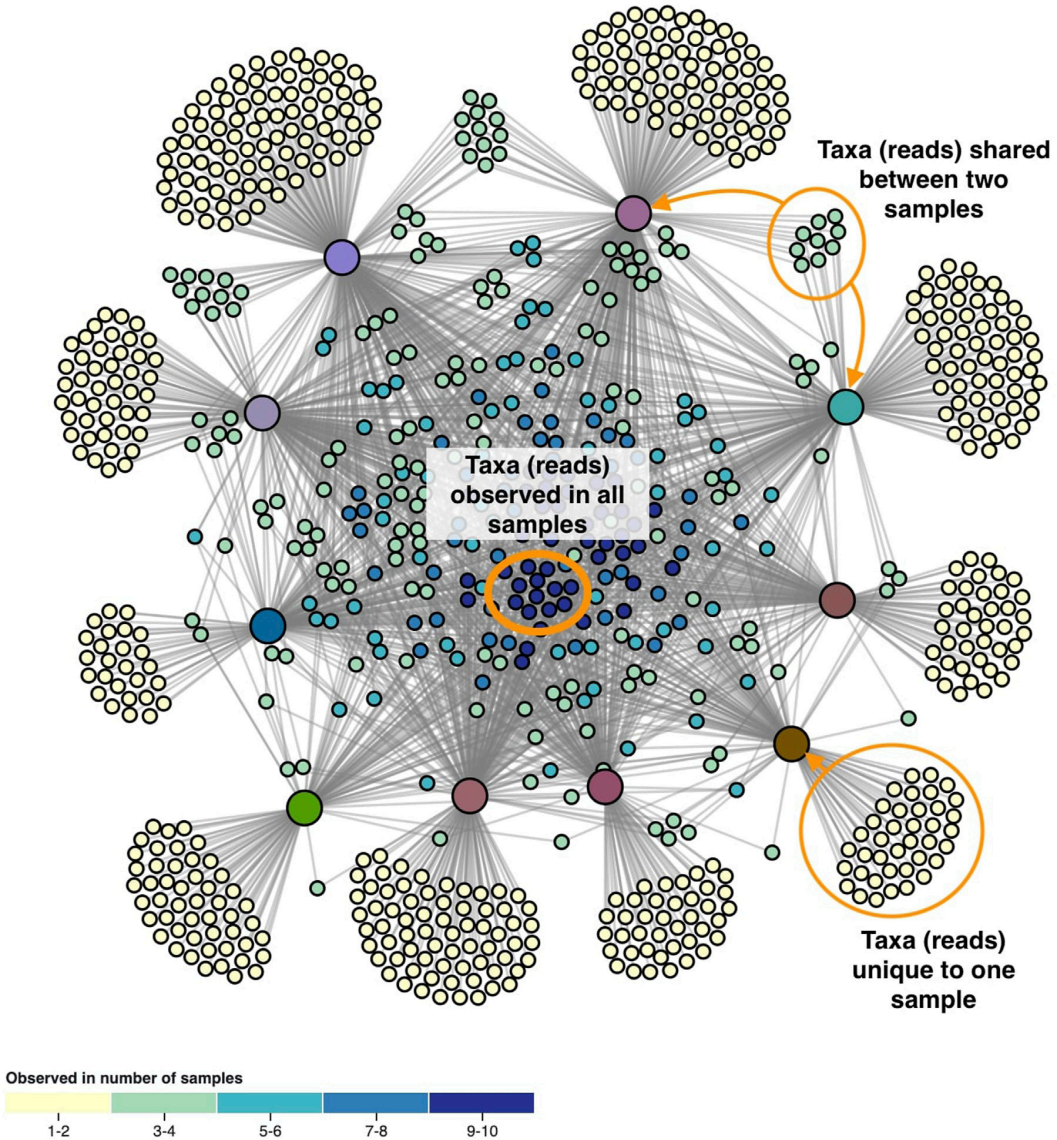
The network’s topology is annotated with the interpretation of the positioning of the ASVs. Samples are colored based on beta diversity and ASVs on the number of samples they are observed in, using the color scales shown in Figure 4. Similarities in microbiome compositions between samples can be identified by the similar coloring.

The layout of the node-link diagram is driven by the network’s topology, using D3’s force-directed layout algorithm (Bostock et al., 2011). All nodes repulse each other by default, but links introduce an attraction force between the nodes they connect. As such the OTUs/ASVs will be positioned in-between the samples they were observed in. This results in a network spatialization driven by the relatedness of the nodes, in which similar nodes tend to be closer to each other. However, this cannot be taken for granted as geometric distance does not apply in the interpretation of topology-driven node-link diagrams (Venturini et al., 2021). A centering force is applied to attract nodes to the middle of the screen, and an additional radial force is applied to the sample and taxa nodes separately to improve the readability. This force pushes sample nodes to the periphery of an imaginary circle and pulls taxa nodes to the center proportional to the number of samples they were observed in. In effect, this force pulls the core microbiome to the center, with less co-appearing taxa surrounding them (Figure 2).

**FIGURE 3:**
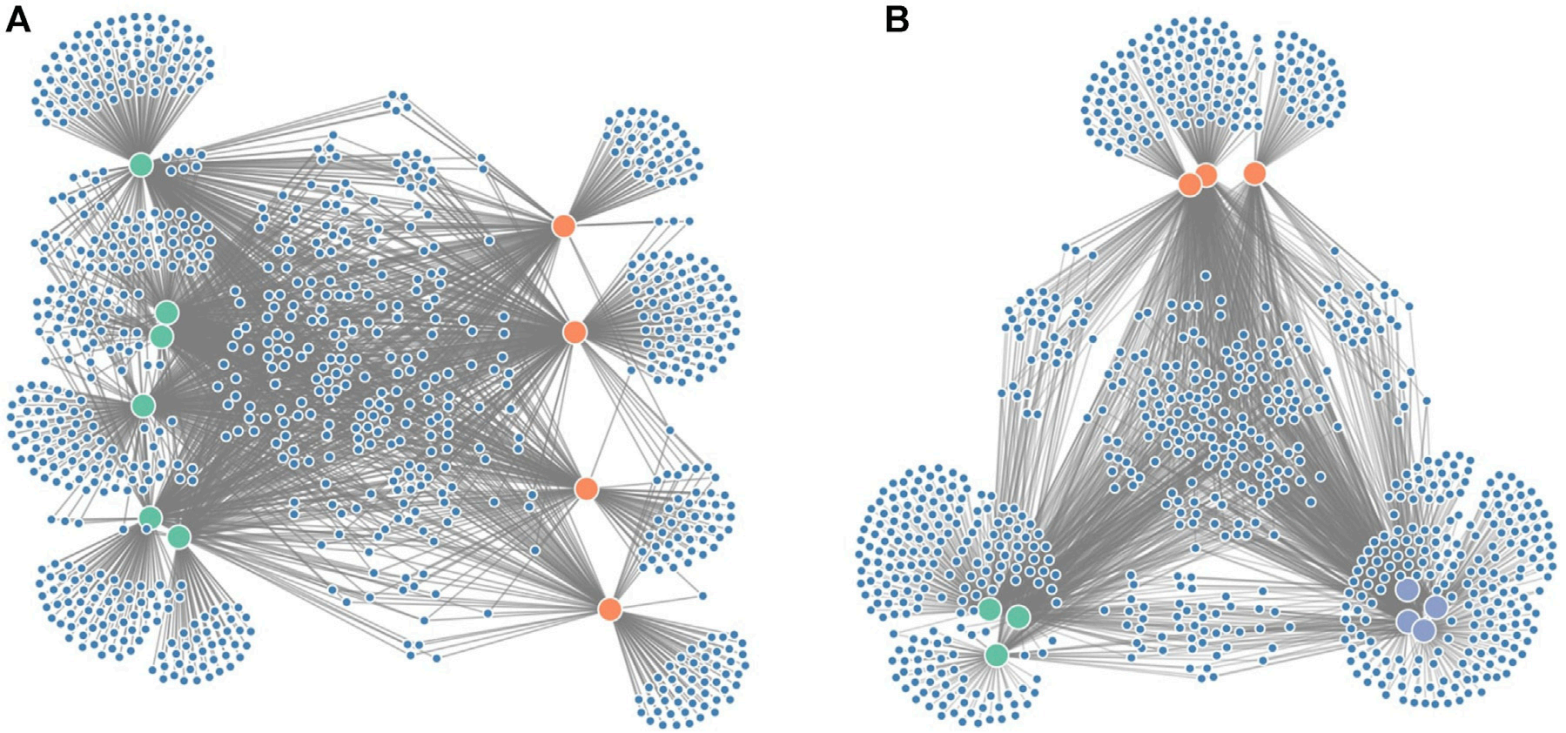
Figure showing the grouping forces applied on the node-link diagram: **(A)** force on the x-axis to distinguish groups of interest (e.g., diseased vs. healthy in a clinical study) and **(B)** force on both the x- and y-axis to distinguish three groups.

Since it is often of interest to compare microbiome compositions between certain groups (e.g., disease vs. healthy), we propose an additional force on the x-coordinates (Figure 3A) to distinguish these groups better. In the case of two groups, one group will be attracted to the left part of the screen, whereas the other group will be attracted to the right part of the screen. If the grouping variable plays an important role in the microbiome composition, we expect sample nodes to be perfectly aligned on their given x-coordinates. One or more samples showing more similarities towards the other group will end up more towards the middle of the network. If more than two groups are to be distinguished, an additional force can be applied to the y-coordinates (Figure 3B).

**FIGURE 4:**
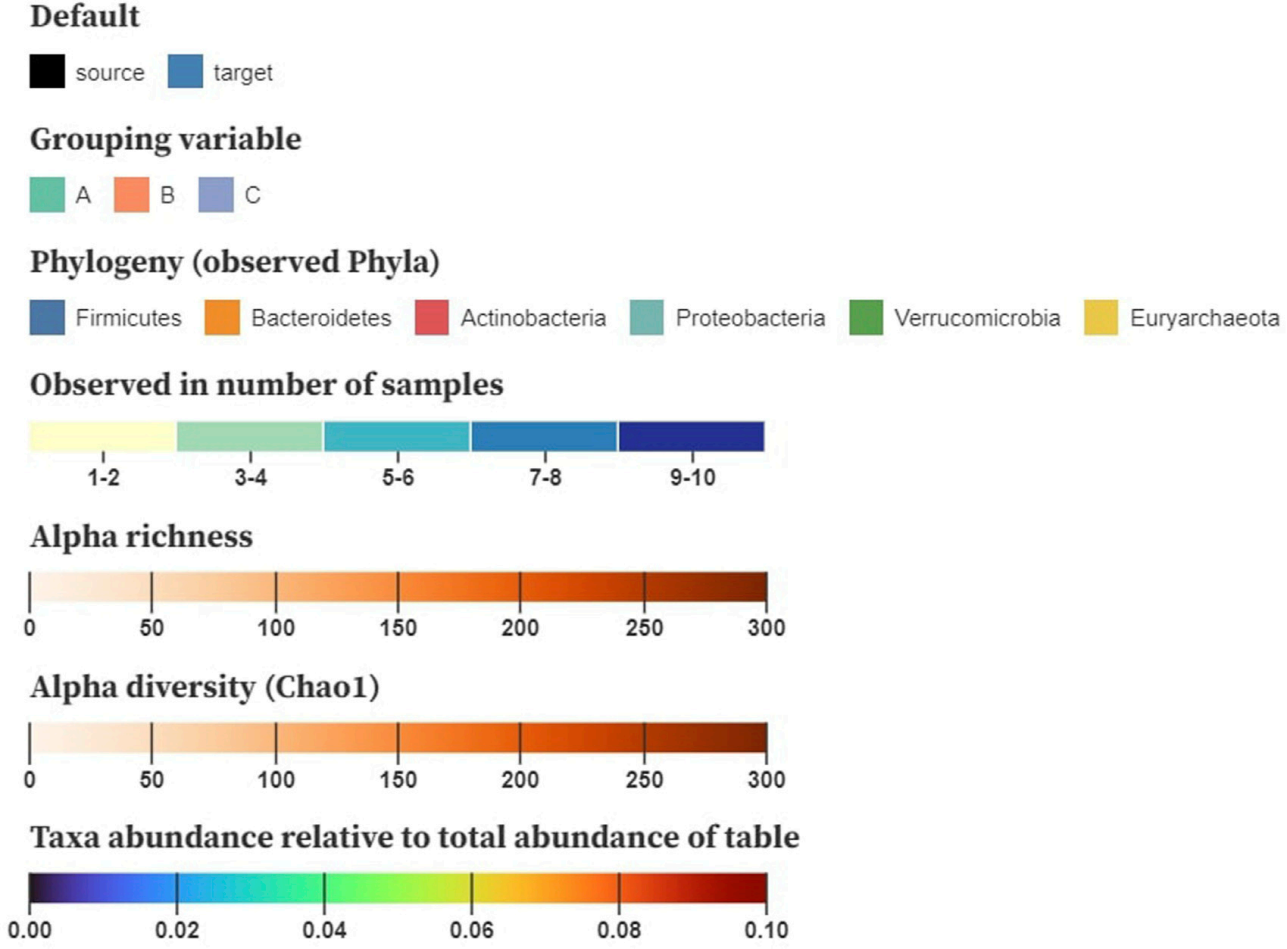
Example color schemes to encode baseline characteristics on the nodes; nominal attributes are shown using a categorical color scale, number of samples a taxon is observed in denoted using multi-hue (binned in intervals), alpha richness and diversity displayed using saturation (single-hue), and taxa abundance relative to the total table abundance shown using sequential multi-hue. Matrices storing pairwise distances, similarities or correlations are mapped to a three-dimensional space using multidimensional scaling (MDS) and translated to CIELAB.

Color is used to encode the node and link attributes in the visualization, for which we base ourselves upon the design principles by Mackinlay (1986). After node position, saturation and hue are the next-best visual channels for encoding quantitative data that can be applied on networks (Nobre et al., 2019). The color encodings we are using are shown in Figure 4. We generally suggest using saturation to display numerical properties (e.g., alpha diversity) and hue for categorical (e.g., disease status), although some exceptions apply. When the difference between the minimum and the maximum value is small (e.g., relative abundance taken over all samples), a combination of both, called a multi-hue scale, can be more informative. When the domain consists of a limited range of integer values (e.g., the number of samples a taxon is observed in), we prefer to bin these values in small intervals to increase perceptual differences.

For downstream analysis that results in matrices (e.g., pairwise distances, similarities, and correlation), inspired by Evers et al. (2021), we propose using multidimensional scaling (MDS) to project the samples to a three-dimensional space. These new dimensions can then be translated to the CIELAB color space, in which a perceived change in color resembles its geometric distance. Hence, similar color values denote samples with a similar composition. As the interpretation of diversity through color relies on the viewer’s perception, it might be cumbersome for people with a lower visual ability (e.g., color blindness). Nonetheless, the CIELAB color space is designed to be perceptually uniform and addresses this problem.

**FIGURE 5:**
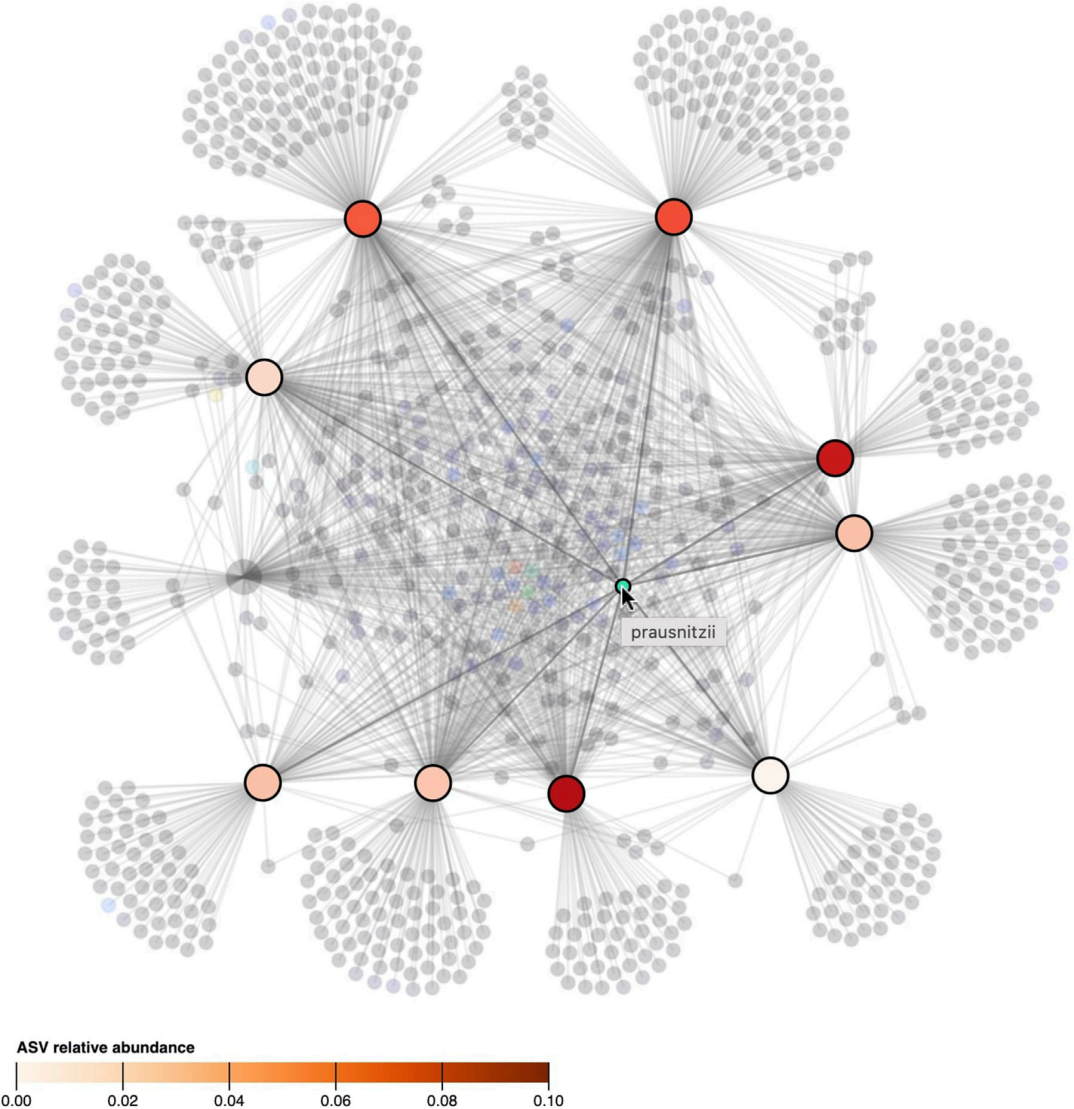
When the cursor hovers over a taxon, its presence, as well as its relative abundance in the samples, is denoted by highlighting and coloring (saturation) of the samples. All other taxa and samples in which the taxon is not observed in are faded. The ASV hovered in the figure, classified as “prausnitzii”, is observed in 9 out of 10 samples but its relative abundance differs between samples.

To represent relational attributes (e.g., relative abundance), we chose again to use color encoding in the nodes rather than in the lines that link samples to taxa as they could overlap. A problem arises when an object should have multiple colors at the same time (i.e., when a taxon is observed in multiple samples and *vice versa*). Hence, our solution is to color the connected nodes based on the link attribute when a taxon or sample is hovered. Figure 5, shows how hovering a taxon highlights the samples it is observed in and colors them based on its relative abundance.

**FIGURE 6:**
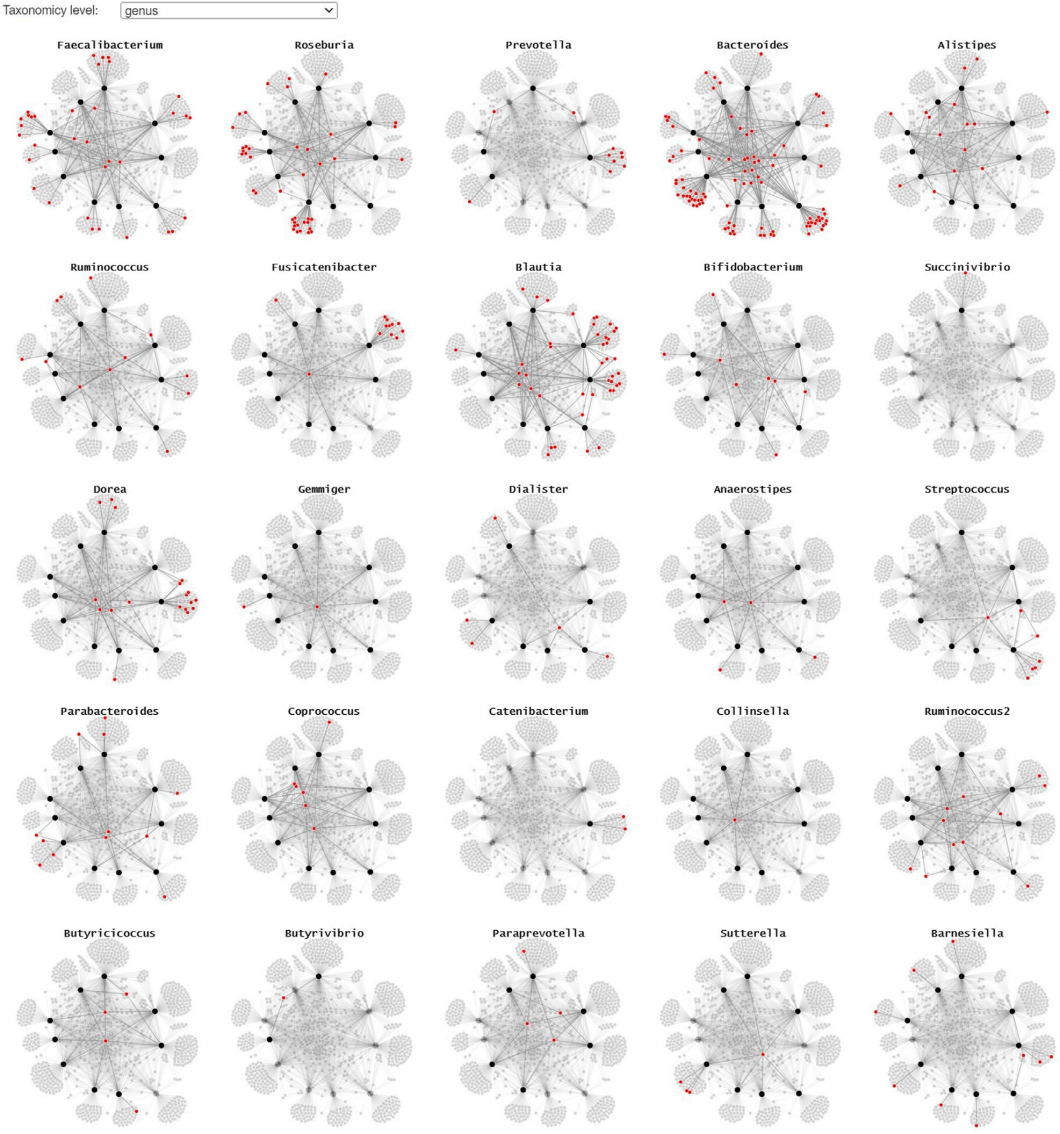
A small multiple representation of the same node-link diagram, highlighting the taxa (genus level) in red. Nodes and links that are not linked to the highlighted genus are faded, providing a clear overview of the samples the taxa are observed in. This representation lends itself well for static/explanatory graphics.

Since the use of color in categorical schemes is limited to a certain number of distinct categories for it to be still readable, we propose two alternative representations to show where the different taxonomies occur in the node-link diagram. By using small multiples with a common layout and highlighting the microorganism belonging to a particular taxonomy in red, we can get a better overview of their presence in the collected samples (Figure 6). This representation lends itself for static presentation, as it provides the overview of all data while at the same time showing the details. If the interest is only in one or a set of specific taxa, one can use a custom color scale in one node-link diagram that highlights all taxa of interest.

#### 2.2.2 Adjacency matrix

**FIGURE 7:**
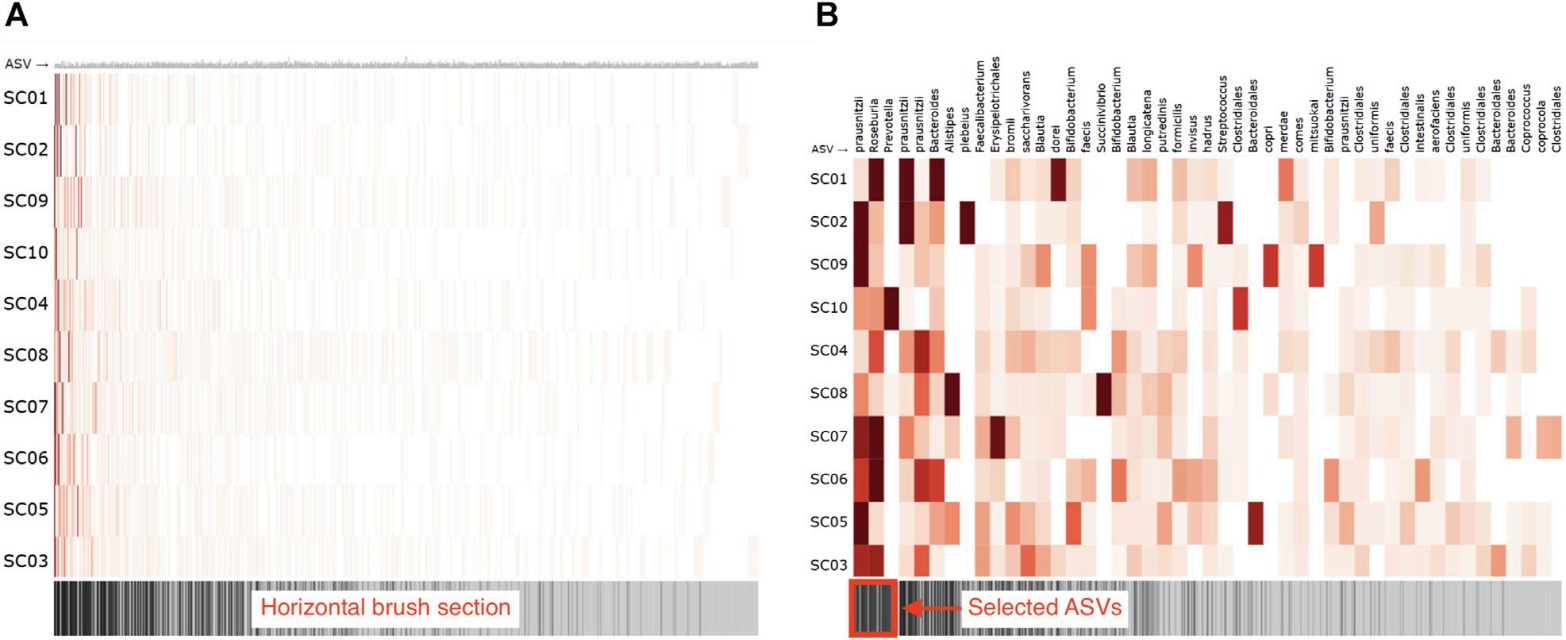
Adjacency matrix showing microbiome abundance data in which relative abundance is denoted in the color saturation. The adjacency matrix is sorted using a seriation algorithm (hierarchical clustering). The barcode below the adjacency matrix denotes the presence of ASVs (i.e., ASVs observed in more samples have darker lines) and acts as a horizontal brush to zoom in on a selected group of ASVs. **(A)** adjacency matrix’s view without using the horizontal brush, and **(B)** using the horizontal brush. See the digital version for best image quality.

To visually represent the microbiome composition graph in an adjacency matrix, samples are shown on the vertical axis, and OTUs/ASVs are shown on the horizontal axis. The relation between the sample and OTU/ASV is denoted in the cells (Figure 7A). One can opt to color all cells for which a relation exists with a fixed color, emphasizing the presence of microbes in a sample, or color them by absolute or relative abundance (weight property). Node attributes can be displayed in or next to their labels (e.g., text coloring). A horizontal brush can be added to zoom in on the OTUs/ASVs (Figure 7B).

An important aspect of an adjacency matrix’s visual clarity is the row and column order. Over the years, much research has been conducted on seriation algorithms that reorder the rows and columns of tabular layouts to reveal higher-order patterns such as clusters and highly connected vertices that might be obscured by the adjacency matrix’s original arrangement (Behrisch et al., 2016). Sakai et al. (2014) describe an ordering method that maximizes the interpretability of global patterns and higher legibility on smaller displays. Berisch et al. (2016) provides an extensive overview of such algorithms and guide toward suitable algorithms for detecting different patterns in networks. The application of these algorithms is essential for understanding a network’s topology and corrects many of the tabular layout’s limitations (Nobre et al., 2019). Although graph-based reordering algorithms do not seem to be applied yet in microbiome research, studies have used hierarchical clustering (Piro and Renard, 2022) or principal component analysis (Fahimipour et al., 2018, 2017) for reordering. In our R package, we rely on the seriation package available in R (Hahsler et al., 2008) and provide a horizontal brush to zoom in on taxa or patterns of interest.

### 2.3 Evaluation

We evaluated Snowflake, specifically focusing on its representation as a node-link diagram, as no novel elements are introduced to the adjacency matrix that has not been assessed before. We conducted the heuristic approach proposed by Wall et al. (2019), which is based on the ICE-T methodology (Stasko, 2014). The ICE-T value equation consists of four key components: Insights (I), Confidence (C), Time (T), and Essence (E). *Insights* focuses on its ability to stimulate insights or provoke insightful questions, while *Confidence* relates to its potential to instill confidence, knowledge, and trust in the data, its domain, and context. *Essence* pertains to the visualization’s capability to convey an overarching understanding of the data, and *Time* refers to a visualization’s capacity to reduce the time required for answering diverse data-related questions. Wall et al. (2019) further deconstruct these components into guidelines, each comprising a set of low-level heuristics. This approach yielded a comprehensive 21-question questionnaire, which was employed to evaluate our method by a group of six domain experts. This number of participants suffices as the authors claim that five raters are adequate to obtain consistent results using their methodology. Each heuristic is rated on a 7-point Likert scale ranging from 1-*strongly disagree* to 7*-strongly agree*, and a visualization can be considered “good” if an overall cumulative average score of 5 or higher is obtained. In the initial terminology of the questionnaire, the terms “data cases” and “data attributes” were used and replaced by the terms “samples” and “ASVs” as the authors noted there were some misconceptions about it. Notes were taken during the evaluation procedure to record evaluators’ feedback.

A user interface (UI) was designed as a proof of principle to enhance the user’s interaction with the visualization method. This UI featured a node-link diagram displaying the microbiome composition graph and a 2D projection of the samples, derived from beta-diversity metrics, to enable users to select a set of samples for further in-depth exploration of their microbial compositions within the node-link diagram. Additionally, one could color the nodes according to the color schemes discussed above and highlight taxa of interest or those demonstrating significant differences in abundance. To initiate the evaluation process, each participant received a concise introduction to the visualization method. Participants were enlightened on transforming an abundance table into a microbiome composition graph. They gained insights into the node-link diagram, with a focus on the distinction between sample and ASV nodes, as well as identifying groupings in terms of connectivity. Additionally, a comprehensive description of the user interface (UI) and the interactions it offered was provided. To facilitate their initial engagement with Snowflake, three data-specific questions were also presented. One is about the identification and location of a specific species in the node-link diagram, one is regarding the grouping of the ASVs and how these groups are related to the selected samples, and the last one is about locating significant differential abundant taxa. Subsequently, participants were afforded 30 min to interact with the visualization method through the UI. During that time, they were encouraged to explore its features and functionalities to complete the evaluation questionnaire.

## 3 Results

The evaluation of our proposed visualization method resulted in an overall cumulative average score of 5.35 (Table 1), with a cumulative average of 5.46 on *insight (I)*, 5.63 on *time (T)*, 5.08 on *essence (E)* and 5.04 on *confidence (C)*. The provided ratings are relatively positive, with no strongly negative assessments. Notably, the visualization was well-received for exposing individual samples and their observed ASVs, generating data-driven questions, and facilitating direct interaction with the data representation. It also effectively highlighted issues related to data quality. Moreover, the visualization was commended for its ability to provide a meaningful spatial organization of the data and its support for smooth transitions between different levels of detail. This suggests that the method effectively helps users organize and navigate complex microbiome data sets, and facilitates higher-level insights and domain knowledge extraction. However, it is noteworthy that for specific questions, such as Q2, Q3, and Q5 (see Table 1), a degree of variation in ratings was observed. This variance may suggest that some respondents had differing perspectives on the visualization method. It is worth considering that individual differences, including varying levels of experience with data visualization, might have influenced these ratings. While the majority of respondents provided favorable feedback, these divergent responses underscore the importance of addressing potential user-specific factors that could impact their understanding and appreciation of the visualization method. Further investigation into the individual factors contributing to these varied responses may aid in tailoring the method to a wider range of users and ensuring its effectiveness, even for those with differing levels of familiarity with visual data analysis. Furthermore, we recognize a few lower scores for Q21, indicating a desire for better handling of potential data issues. A possible fix could be to communicate the number of missing or incorrect values at the return of the data transformation.

**TABLE 1:** Results obtained from the evaluation by a group of six domain experts. An overall cumulative average of 5.35 is received.

	Question	R1	R2	R3	R4	R5	R6	Average
Insight	Q1: The visualization exposes individual samples and their observed ASVs	7	7	6	6	7	7	6.67
	Q2: The visualization facilitates perceiving relationships in the data like patterns of the variables	5	6	3	5	6	6	5.17
	Q3: The visualization promotes exploring relationships between individual samples as well as different groupings of samples	7	4	2	6	4	6	4.83
	Q4: The visualization helps generate data-driven questions	7	6	6	5	6	6	6
	Q5: The visualization helps identify unusual or unexpected, yet valid, data characteristics or values	6	6	4	6	6	5	5.5
	Q6: The visualization provides useful interactive capabilities to help investigate the data in multiple ways	5	7	2	6	5	7	5.33
	Q7: The visualization shows multiple perspectives about the data	5	5	3	4	5	5	4.5
	Q8: The visualization uses an effective representation of the data that shows related and partially related samples	4	7	5	6	6	6	5.67
Time	Q9: The visualization provides a meaningful spatial organization of the data	4	7	5	6	6	5	5.5
	Q10: The visualization shows key characteristics of the data at a glance	5	4	3	6	4	7	4.83
	Q11: The interface supports using different attributes of the data to organize the visualization’s appearance	6	6	3	6	5	7	5.5
	Q12: The visualization supports smooth transitions between different levels of detail in viewing the data	5	6	6	5	5	6	5.5
	Q13: The visualization avoids complex commands and textual queries by providing direct interaction with the data representation	7	7	7	7	6	7	6.83
Essence	Q14: The visualization provides a comprehensive and accessible overview of the data	6	5	1	6	5	5	4.67
	Q15: The visualization presents the data by providing a meaningful visual schema	6	5	3	6	6	6	5.33
	Q16: The visualization facilitates generalizations and extrapolations of patterns and conclusions	4	6	5	5	4	5	4.83
	Q17: The visualization helps understand how variables relate in order to accomplish different analytic tasks	5	6	5	6	5	6	5.5
Confidence	Q18: The visualization uses meaningful and accurate visual encodings to represent the data	5	6	5	6	7	6	5.83
	Q19: The visualization avoids using misleading representations	7	3	3	4	6	5	4.67
	Q20: The visualization promotes understanding data domain characteristics beyond the individual samples and ASVs	7	4	6	5	5	6	5.5
	Q21: If there were data issues like unexpected, duplicate, missing, or invalid data, the visualization would highlight those issues	3	4	6	3	4	5	4.17

Nonetheless, we can conclude that Snowflake passes the evaluation taken by our group of domain experts since an average score above 5 was obtained. Their comments indicate our method allows user-friendly exploration of the microbiome and its composition. They especially appreciated the fact that every read is visible, and aggregations in the taxonomic classification are not required, although they can be done. The fact that this method allows users to include all results from downstream analysis in the node attributes makes it possible to relate them to the composition and the spatialization of the network. From an immunological perspective, changing the spatialization of the network based on a grouping variable is considered to facilitate the identification of candidate taxa. The inclusion of some additional features and interactions for the drawing of, and capturing information from, the microbiome composition graph was proposed by some of the participants. Several participants mentioned they were eager to apply this visualization method to their own data.

## 4 Discussion

This paper introduces Snowflake, a new visualization method to visualize microbiome abundance tables using multivariate bipartite graphs. Unlike conventional microbiome visualization methods (e.g., stacked barcharts and tree-structured visualizations), in our design, every observed OTU/ASV in the microbiome abundance table is visually encoded such that no information is lost due to aggregation in taxonomic classifications or neglecting less abundant reads. The topological structure of the data allows users to get a visual overview of the microbiome composition in the collected samples. We used a node-link diagram to represent the microbiome composition graph and provide an alternative representation using an adjacency matrix. Using “on-node encoding,” the appearance of the node-link diagram can be changed based on additional information stored in the node attributes. This includes, but is not limited to, the hierarchical structure of the data and any other information obtained from downstream analysis (e.g., alpha-and beta-diversity) and metadata. We suggest an appropriate color encoding for every type of node and link attribute.

Following an evaluation study conducted with domain experts, our visualization method has been deemed promising for the visual exploration of microbiome data. With our visual representation, we show that our method can be used to easily identify which microorganisms are unique to certain samples and which are common among multiple samples. This can provide experts with insight into the core microbiome, and help identify candidate microorganisms shared between certain sample cohorts in clinical studies. By showing the microbiome composition graph as a node-link diagram, differences between samples in terms of composition, richness, and diversity can conveniently be found by looking at the topological structure. By means of on-node encoding—i.e., changing the appearance of nodes in a network—using color hue and saturation, we present a solution for all types of additional node and link attributes to be displayed in the visuals.

As the dataset size expands, leading to an increased number of samples and taxa to be represented in the node-link diagram, our method may face challenges. These challenges encompass potential issues in the computational efficiency of the force-directed layout algorithm and difficulties arising from occlusion, specifically caused by numerous overlapping links within the node-link diagram. Therefore, our visualization method is designed with a focus on in-depth data exploration. While concerns about scalability for large datasets are valid, our intended use of this visualization method is to provide a powerful tool for users to delve into data with precision. The primary objective is to enable users to select a specific group of samples and examine them in intricate detail, facilitating a comprehensive comparison of their composition in relation to each other. This targeted use case ensures that the method excels in delivering insights and valuable information when dealing with smaller, carefully chosen subsets of data. Within our R package, we provide the option to work with the adjacency matrix and node-link diagram in an interactive way. When plotting both visuals at once using the crosstalk R package, the user will be able to make selections within the adjacency matrix that will update the node-link diagram to take a closer look into the selection made. Hence, the user can use the adjacency matrix to zoom in on isolated sub-parts of the graph in the node-link diagram, including one or more samples by clicking on the sample labels. This technique is not new and has been applied in tools such as NodeTrix (Henry et al., 2007) and MOBS (Heylen et al., 2022).

In conclusion, with Snowflake we introduce a method for visualizing microbiome abundance tables using multivariate bipartite graphs. Unlike traditional microbiome visualization techniques, Snowflake maintains the individuality of observed microorganisms without aggregating them into taxonomic classifications. This approach provides a comprehensive overview of the microbiome’s composition in collected samples. Evaluation by domain experts validates Snowflake’s potential for exploring microbiome data, including identifying unique and common microorganisms among samples. The method is tailored for in-depth data exploration, focusing on precision rather than scalability, making it valuable for detailed analysis of carefully selected subsets of data.

## Data Availability

The datasets presented in this study can be found in online repositories. The names of the repository/repositories and accession number(s) can be found in the article/Supplementary Material.
